# Second ever reported case of central cause of unilateral foot drop due to cervical disc herniation: Case report and review of literature

**DOI:** 10.1016/j.ijscr.2021.105928

**Published:** 2021-04-29

**Authors:** Mutaz Alhardallo, Walid El Ansari, Abdul Moeen Baco

**Affiliations:** a Department of Orthopedic Surgery, Hamad General Hospital, Hamad Medical Corporation, Doha, Qatar; b Department of Surgery, Hamad General Hospital, Doha, Qatar; c College of Medicine, Qatar University, Doha, Qatar; d School of Health and Education, University of Skövde, Skövde, Sweden

**Keywords:** Case report, Cervical disc, Foot drop, Upper motor neuron, Anterior cervical decompression and fusion

## Abstract

**Background:**

Foot drop is defined as a weakness in the ankle and foot dorsiflexors. A disruption of the neural pathway starting from the motor prefrontal cortex and ending in the peroneal nerve can lead to foot drop. Foot drop due to lower motor neuron injury is well documented. However, foot drop due to a central cause of cervical disc prolapse is very rare.

**Case presentation:**

A 55-year-old male presenting with neck pain, right and left arms radicular pain and numbness, and unilateral right foot drop following cervical disc prolapse. The patient presented with upper motor neuron lesion signs. MRI showed cervical disc prolapse at two levels, confirming central cause of foot drop. The patient underwent anterior cervical decompression and fusion surgery.

**Discussion:**

Following decompression and fusion of involved cervical spine disc pathology, the patient had complete recovery of his right foot drop.

**Conclusions:**

Central causes, although rare, should be considered in the differential diagnosis of foot drop. Causes could be due to the compression effect of the cortico-spinal tract of the cervical spinal cord. Satisfactory results can be achieved upon correcting the causative lesion.

## Highlights

• Foot drop due to lower motor neuron injury is documented. Foot drop due to central cause cervical disc prolapse is very rare
• 55-year-old male with neck pain and upper motor neuron lesion signs. MRI shows cervical disc prolapse of 2 level, confirming central cause of foot drop
• Central causes are rare but however should be considered in the differential diagnosis of foot drop

## 1 Background

Foot drop (FD) is defined as a weak tibialis anterior (TA) muscle and is often accompanied by weakness of the extension of the toes [1]. Clinically, it starts from a muscle strength manual test result of less than 3/5, i.e., when the foot can no longer be actively lifted against gravity [2].

FD is commonly due to peripheral causes comprising peroneal nerve neuropathy [3,4] due to hereditary or acquired causes e.g., distal myopathies, lower motor neuron disease, surgical nerve trauma, or peroneal nerve entrapment syndromes due to masses causing pressure near the fibular neck where the common peroneal nerve is covered only by skin and subcutaneous tissue [5,6]. Other peripheral causes of FD include L4–L5 radiculopathy caused by degenerative disc disease e.g., posterior-lateral herniated nucleus pulposus affecting the traversing nerve root or foraminal stenosis affecting the exiting nerve root [3].

Whilst the peripheral etiology of FD is well recognized [6], however, FD due to central etiology is very rare [3]. Isolated FD caused by a central upper motor neuron (UMN) insult is scarce and sporadic, unless muscle group weakness co-exists with the FD, as reported in 52% to 67% of patients with spinal UMN pathology [7].

To the best of our knowledge, in terms of central cervical etiology of FD, only one case of unilateral FD caused by a cervical spine lesion has been reported to date [3]. Central etiologies of FD are due to brain lesions [5,8].

We present a 55-year-old African male presenting with two degenerative discs prolapse C5-C6 and C6-C7 as a rare cause of unilateral FD. We report this case in line with the updated consensus-based surgical case report (SCARE) guidelines [9]. In addition, we undertook a literature review of FD due to central causes.

## 2 Case presentation

A 55-year-old African man presented to our Orthopedic Spine Outpatient clinic at our institution at the Bone and Joint Institute of Hamad General Hospital (largest tertiary care facility in Doha, Qatar) with a chief complaint of neck pain since 5 months and weakness of his right foot in the last 2 weeks. History revealed that the neck pain was aggravated with any neck bending activities, and associated with pain and numbness radiating to right arm up to the index and middle finger. Bowel and bladder function were normal, and there was no myelopathic symptoms. Two weeks prior to this presentation, the patient had developed right lower limb radicular pain, numbness, and weakness of the right foot, with no symptoms of back pain. His general medical history included hypertension diagnosed at the age of 50 and treated with medications. The patient is a non-smoker, and no other significant diseases were noted in his general medical history. There was no relevant past surgical or family history.

Upon clinical neuro-examination, both upper limbs had normal tone, and the power on the right upper limb was grade 3/5 Medical Research Council (MRC) [10] for C7 myotomes. There was subjective decreased sensation of pain and light touch for the right and left C6 and C7 dermatomes, increased right C7 hyperflexia, positive Spurling test, negative Lhermitte sign, and negative shoulder abduction test.

Neuro-examination of the lower limbs showed that the patient had a right high steppage gait and normal tone bilaterally. On the right side, the power of right dorsiflexion was 2/5, extensor hallucis longus was 2/5, and plantar flexion was 5/5. The power of the other muscle groups of both lower limbs was normal. The ankle reflex was normal bilaterally, and right knee reflex was 3+. Babinski's test was positive on the right side but negative on the left side. Sensation to pain and light touch was declined on the right L4 and L5 dermatomes, and the straight leg raising test was negative.

**Fig. 1:**
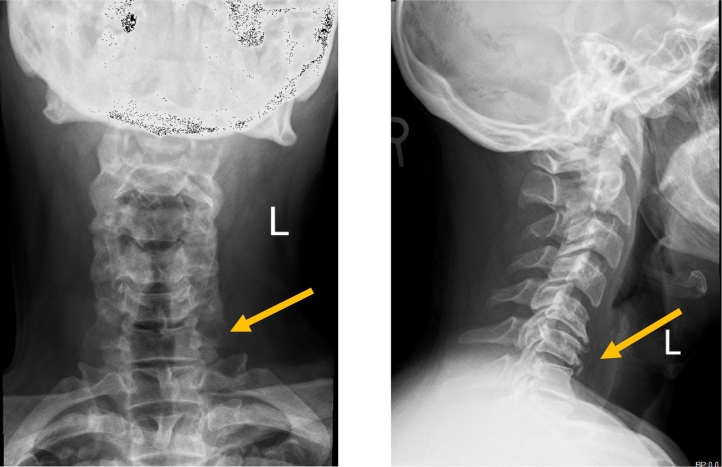
X-ray antero-posterior and lateral views of cervical spine showing C5–C6 and C6–C7 anterior and posterior vertebral osteophyte formation with loss of disc height, as well as kyphotic deformity of cervical spine.

**Fig. 2:**
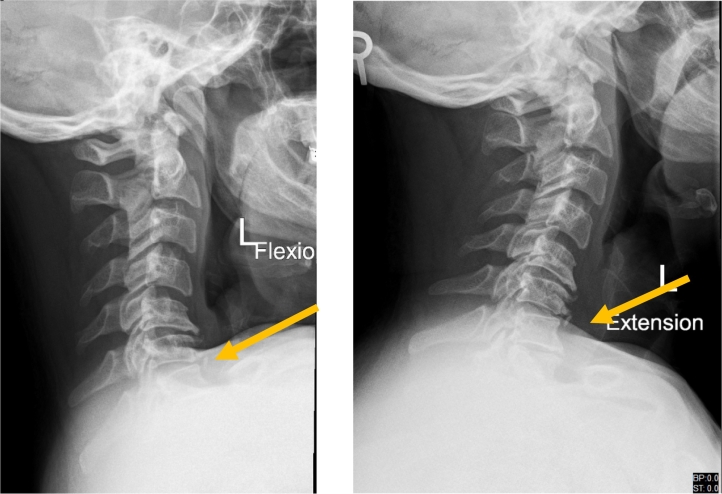
X-ray cervical spine dynamic lateral view showing stable cervical vertebral spine.

**Fig. 3:**
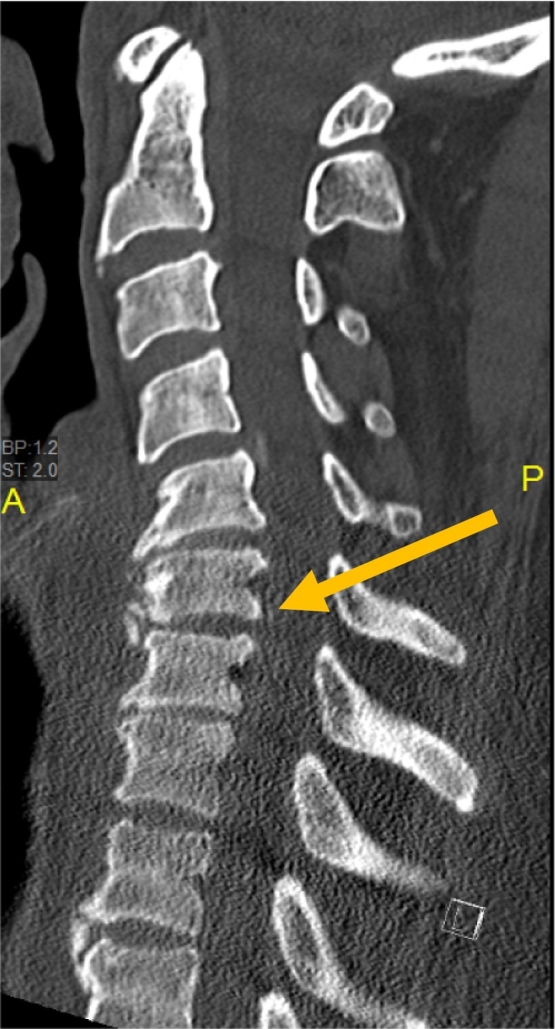
CT scan of cervical spine (sagittal view) showing no calcified posterior longitudinal ligament.

**Fig. 4:**
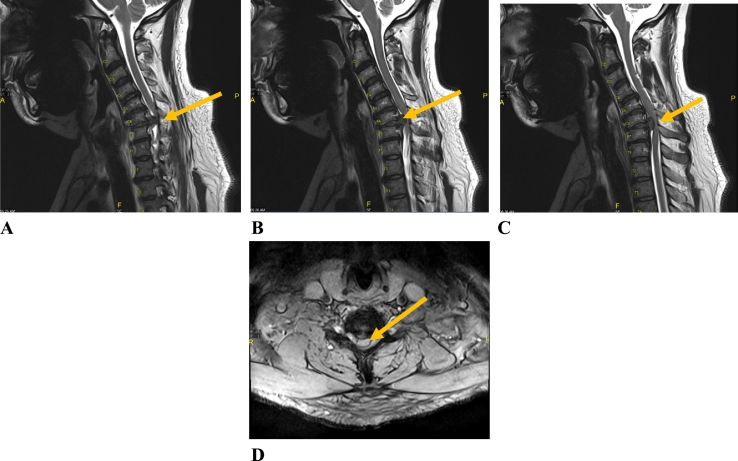
MRI of the cervical spine (lateral view, A, B and C) and C6–C7 (axial view, D) showing significant disc herniation at two levels (C5–C6 and C6–C7) with no spinal cord myelomalacia.

**Fig. 5:**
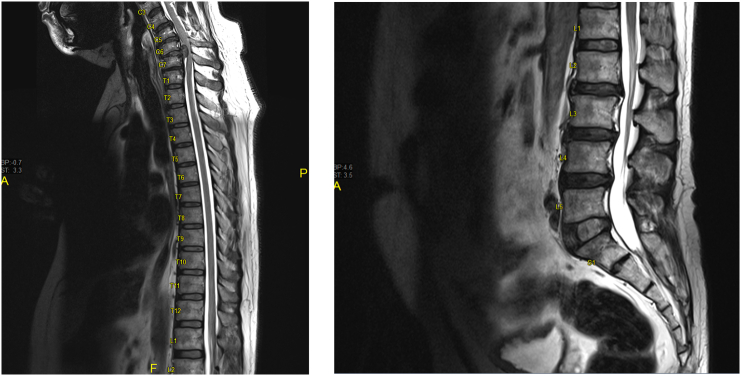
MRI thoracic and lumbar spine (lateral view) showing no major disc herniation or nerve root impingement.

Neuroimaging was performed. X-ray of the cervical spine showed multiple vertebral osteophytes as well as disc degeneration, mainly at the C5-C6 and C6-C7 levels, with kyphotic deformity (Fig. 1) and stable cervical vertebrae (Fig. 2). CT scan depicted no calcified posterior longitudinal ligament (Fig. 3). Magnetic resonance imaging (MRI) of T2 showed significant disc herniation encroaching on the spinal canal mainly at the C5-C6 and C6-C7 levels, with no spinal cord signal changes (Fig. 4). MRI of the lumbar and thoracic spine revealed mild disc degeneration at L3–L4 and L4–L5 (Fig. 5), with no major disc herniation or nerve root impingement, which seemed to be inconsistent with the degree of clinical presentation of the FD.

**Fig. 6:**
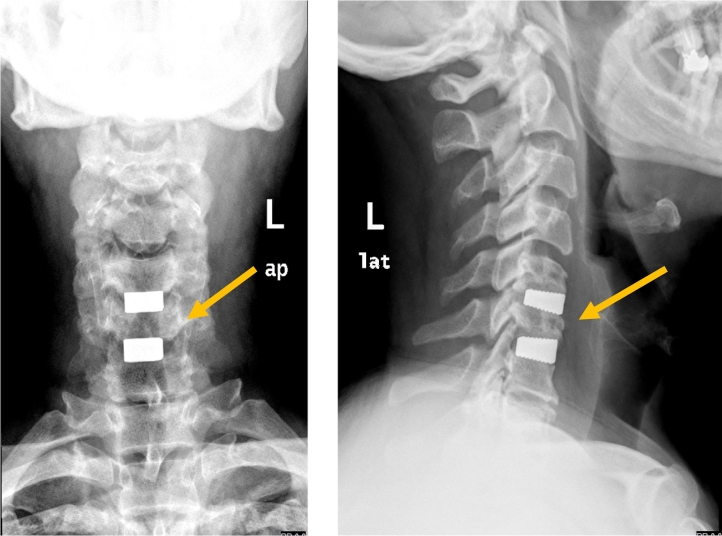
X-ray cervical spine antero-posterior and lateral views showing anterior cervical decompression and fusion at C5–C6 and C6–C7.

The decision was to undertake anterior cervical decompression and fusion (ACDF) at C5–6 and C6–7. Four days after the initial presentation at our clinic, the procedure was undertaken by an experienced consultant spinal surgeon under general anesthesia. The procedure comprised a right sided anterior approach of cervical spine, C5–6 and C6–7 discectomy and interbody fusion using trabecular stand-alone cages, and insertion of wound drain that was kept for 24 h. There were no intra or postoperative complications, and two days postoperatively, the patient had complete and dramatic recovery of his right foot drop. He was discharged on postoperative day 3. The follow up course went smoothly, and follow up x ray was satisfactory (Fig. 6). The patient was followed up for one year. He resumed his normal ordinary activity with satisfaction.

## 3 Discussion

Injury to the neurological pathway that enables ankle dorsiflexion may lead to FD. This includes the brain, the spinal cord, nerve roots, lumbosacral plexus, and sciatic and peroneal nerves [11]. Peripheral neuropathies are the most common causes of FD [1], where patients usually present with lower motor neuron findings on clinical evaluation (e.g., decreased deep tendon reflexes, muscle hypotonia). In contrast, when upper motor neuron findings are present (e.g., increased muscle tone, brisk deep tendon reflex, clonus, positive Babinski's sign), a central lesion should be suspected and appropriate imaging studies are performed to confirm [12,13].

Most of the published FD cases due to central etiologies are due to brain lesions, including cerebral infarction, intra-cranial tumor, traumatic brain injury and multiple sclerosis [5,8]. Some articles reported that thoracic spine disc protrusion cases with FD could be due to UMNL [[14], [15], [16]]. However, our literature review found only one reported case of unilateral central FD was due to cervical pathology [3] (Table 1), with which we are unable to directly compare our findings precisely.

**Table 1:** Literature review of case reports of central causes of foot drop due to cervical disc herniation.

Report	Age/sex	Pathology	Level	Additional signs and symptoms	Laterality	EMG	Treatment
Current Case 2018 Qatar	55/M	Spinal stenosis	C5–C6 and C6–C7	3+ DTR right patella and right triceps, positive Babinski's	Right	Not done	ACDF
Westhout 2007 USA [3]	46/M	Spinal stenosis	C4–C7 and T11–T12	3+ DTR left patella	Bilateral	Done, showed Positive sharp waves	Decompression laminectomy

M: male F: female C: cervical vertebrae: DTR: deep tendon reflex; ACDF: anterior cervical decompression and fusion.

A retrospective investigation of the surgical treatment of 25 patients with FD due to single-level T10- L1 disc protrusion/s that presented with UMNL signs reported that all cases had excellent recovery [14]. Another case study demonstrated the role of surgical therapy in the treatment of spasticity related to cervical spondylosis also reported improvement [17].

The somatic organization of the motor neurons for the lower extremity are located on the medial homunculus of the primary motor cortex at the precentral gyrus, and neuronal tracts go down through the internal capsule to the ventral grey matter of the spinal cord as cortico-spinal tract; an insult to this circuit due to ischemia or compression may lead to FD [2,18].

The diagnosis of the central etiology for FD might be delayed in the absence of clear signs of upper motor neuron involvement [2]. In our case, careful neurological examination prompted us to suspect central disease (hyper-reflexia of right triceps and right patella deep tendon reflexes, positive right Babinski's sign). However, as FD is commonly caused by peripheral neuropathy, we accordingly undertook MRI of the lumbar and thoracic spine despite the lack of lumbar and thoracic spine symptoms, in order to rule out possible and common FD etiologies. we attributed the possible cause of FD to the fact that a cervical disc prolapse could affect the pyramidal tract of the leg which is located more lateral at the cervical spinal cord [3]. Dissociated symptoms may indicate central UMN pathology in the spinal cord or brain as opposed to peripheral nerve or nerve root, as has been proposed by others for a patient with bilateral FD [3].

We did not undertake electromyography of the TA muscle. Recovery of evoked muscle action potentials of the TA is known to be very late and does not occur at the same time as the recovery from the symptom of FD [14]. Our patient demonstrated clinical recovery from the FD after management (Table 1).

## 4 Conclusions

Although the most common cause of FD is peripheral nervous system disorder, rare central nervous system lesions should always be suspected and considered. The presence of upper motor neuron findings such as positive Babinski's sign, hyper-reflexia or clonus in patients with FD should alert the clinician to such possibility. Cervical disc prolapse could present with FD due to compression of cortico-spinal tract. MRI could confirm or refute such suspicion. Correction of such a central cause of FD by decompression results in a dramatic and swift recovery. Central causes of FD must be included in the workup once peripheral and radiculopathic causes have been ruled out.

## Sources of funding

Nothing to declare.

## Ethical approval

Approved by Medical Research Center, Hamad Medical Corporation reference number (MRC 04-21-201).

## Consent

It was not possible to obtain written informed consent from the patient. The head of our medical team has taken responsibility that exhaustive attempts have been made to contact the family and that the paper has been sufficiently anonymized not to cause harm to the patient or his family. A copy of a signed document stating this is available for review by the Editor-in-Chief of this journal on request.

## CRediT authorship contribution statement

**Mutaz A. Alhardallo**: Conceptualization, Data curation, Investigation, Writing -review & editing. **Walid El Ansari**: Conceptualization, Data curation, Investigation, Methodology, Project administration, Writing - review & editing. **Abdalmoeen Baco**: Data curation, Investigation, Methodology, Writing - review & editing. All authors critically reviewed, revised and contributed to the final article. All authors read and approved the final manuscript.

## Guarantor

Prof Dr. Walid El Ansari: welansari9@gmail.com.

## Provenance and peer review

Not commissioned, externally peer-reviewed.

## Registration of research studies

Not first in Man, hence UIN not required.

## Declaration of competing interest

Nothing to declare.
